# Mass variability in drops of multidose eyedrops: a quality and reliability study

**DOI:** 10.5935/0004-2749.20210096

**Published:** 2025-08-21

**Authors:** Alexandre Xavier da Costa, Priscila Cardoso Cristovam, Joyce Luciana Covre, Maria Cecilia, Zorat Yu, Lauren Carmela LaMonica, José Álvaro Pereira Gomes, Vagner Rogério dos Santos

**Affiliations:** 1 Department of Ophthalmology and Visual Science, Escola Paulista de Medicina, Universidade Federal de São Paulo, São Paulo, SP, Brazil; 2 Yale School of Public Health, New Haven, CT, USA

**Keywords:** Ophthalmic solutions/administration & dosage, Reference pattern of eyedrops, Multidose eyedrop standardization, Ophthalmic drug delivery, Quality, Soluções oftálmicas/administração & dosagem, Volume da gota, Padrão de referência dos colírios, Padronização de colírios multidose, Administração de medicamentos oftálmicos, Qualidade

## Abstract

**Purpose:**

This quality and reliability study aimed to identify the mass variability of multidose eyedrops and to verify the existence of a reference pattern for the drop volume of eyedrops using standard lubricant eyedrops available on the Brazilian market.

**Methods:**

Five brands of lubricant eyedrops were evaluated. An ideal standard 20 µL drop of eyedrops from each manufacturer was captured using an adjustable micropipette. The eyedrop bottles were randomly selected, and five measurements of the samples’ masses were collected using calibrated precision scales.

**Results:**

The mass of the 20 µL samples varied significantly (p<0.001) among the different manufacturers. However, among eyedrops of the same brand, mass variation was not statistically different. The global mean mass of all weighed drops was 18.24 mg, and non-uniformity was identified across all eyedrop brands.

**Conclusion:**

Significant variations in the drop masses of common lubricant eyedrops were identified using standard laboratory equipment. Heterogeneity in the drop volume of standard eyedrop medications suggest that potential dosage discrepancies exist, possibly altering treatment efficacy. A pre-established reference measure may lead to the production of more appropriately sized eyedrops for use in human eyes.

## Abstract (pt)

**Objetivo:**

Este estudo de qualidade e confiabilidade teve como objetivo identificar a variabilidade da massa do volume da gota de colírios multidose e verificar a existência de um padrão de referência para o volume da gota dos colírios usando lágrimas artificiais do mercado brasileiro.

**Métodos:**

Cinco marcas de colírios lubrificantes foram avaliadas quanto ao volume da gota. Uma gota padrão ideal de 20 µL de cada fabricante foi coletada usando uma micropipeta ajustável. Os frascos dos colírios foram selecionados aleatoriamente e cinco medidas das massas das amostras foram coletadas usando escalas de precisão calibradas.

**Resultados:**

A massa das amostras de 20 µL variou significativamente (p<0,001) entre os diferentes fabricantes. No entanto, entre os colírios da mesma marca, a variação da massa não foi estatisticamente diferente. A massa média global de todas as gotas pesadas foi de 18,24 mg e foi observada uma não-uniformidade entre todas as marcas de colírios.

**Conclusão:**

Identificou-se uma variação significativa nas massas do volume das gotas dos colírios lubrificantes, usando equipamento padrão de laboratório. A heterogeneidade no volume da gota dos colírios testados sugere a existência de discrepâncias potenciais em suas posologias, possivelmente alterando a eficácia do tratamento. Uma medida de referência pré-estabelecida pode levar à produção de colírios com gotas de tamanho mais apropriado para uso em olhos humanos.

## INTRODUCTION

Eyedrops are widely used as a unit of measurement for drug delivery, but a considerable degree of variation may exist in the dose administered^(1)^. Variation in doses of commercial eyedrops is often accompanied by adverse side effects, of which systemic toxicity is one of the most prominent and threatening to patients^(2)^. Despite the general belief that most topical medications with dropper bottles maintain a constant ratio of drops per mL, eyedrops often do not have a consistent mass or volume due to several intervening factors.^(3,4)^ These factors include the drug formulation, the physicochemical characteristics of the solution or bottle, the concentration of the solution, the ambient pressure and temperature, and the drop bottle geometry, all of which may contribute to a lack of drop dosage uniformity^(1-5)^.

The Brazilian Pharmacopoeia is the official pharmaceutical code followed in Brazil and defines the standards and specifications of pharmaceutical inputs, medicine, and other products that are subject to sanitary surveillance^(6)^. In a previous edition^(7)^, the Brazilian Pharmacopoeia followed the specifications of the current American Pharmacopoeia and defined that any measuring instrument for the administration of liquid medicine should meet specific volumetric standards: an official medicine dropper must have an outside diameter of 3 mm and dispense 20 drops of distilled water per mL at a temperature of 25°C with an expected weight of 1 g^(7,8)^. The volume of each drop should therefore equate to 50 µL on average, a measure cited as the maximum value allowed by the Brazilian Ministry of Health for the drop volume of eyedrops^(9,10)^. However, the drop volume of multidose eyedrops in Brazil varies widely, ranging from an average of 25.6 µL in Octifen^®^ (ketotifen fumarate 0.25 mg/mL; 39 drops/mL)^(11)^ to 71.4 µL in Trisorb^®^ (dextran 70 1 mg/mL + hypromellose 3 mg/mL + glycerol 2 mg/mL; 14 drops/mL)^(12)^.

In 2010, the Fifth Edition of the Brazilian Pharmacopoeia introduced the ‘Dripping Test’, which is the first suggested standardization method for verifying the ratio of the number of drops per mL and the amount of drug per drop in liquid dosage forms. Drops per mL can be calculated by weighing 20 drops of the product on a precision scale, multiplying by the density of the solution used, and dividing by the measured mass of the 20 drops^(13)^. This method allows for the calculation of the mean number of drops that corresponds to 1 mL of a solution and, subsequently, the average volume of each drop. However, current Brazilian legislation no longer defines general specifications for the volume of drops. Moreover, regulations for eyedrop dosage are lacking globally; in fact, neither the Medicines Control Agency in the UK nor the Food and Drug Administration in the USA have current specifications regarding standardized drop volume^(1)^.

The lack of drop volume specifications have led to an overlooked problem involving the maximal volume of an eyedrop for topical use. The drop volume of commercial drop dispensers often significantly exceeds the limited capacity of the conjunctival sac^(14)^, leading to drainage out of the eye and, in some cases, adverse side effects, including hyperpigmentation of the eyelid, skin irritation, or allergy, as well as systemic absorption of the drug due to increased flow through the lacrimal pathways^(15-18)^. As a consequence of these potential adverse side effects, it has been suggested that an ideal drop for the human eye should not exceed 20 µL^(19)^. Additionally, specifications do not account for confounding factors, such as the viscosity and density of the ophthalmic solution, the packaging of solutions in different bottles by various manufacturers, or dosage instillation and adherence, which should all be considered as important intervening factors^(4,10,20)^.

Establishing a reference during the process of quality evaluation is critical because it guides comparative studies of proper eyedrop dosage in ophthalmic practice. Propagation of any error may influence the process of evaluating and validating drug delivery from various eyedrop dispensing systems^(21)^. Despite extensive quality control checks that are routinely conducted on the content of ophthalmic drugs, few studies have comprehensively addressed this variation in eyedrop volume^(22,23)^. The purpose of this study is to evaluate the variability in the mass of 20 µL samples and to verify the existence of a reference pattern for the drop volume of eyedrops.

## METHODS

This is a laboratory study performed in the Advanced Center of Ocular Surface of the *Escola Paulista de Medicina da Universidade Federal de São Paulo*. The study was approved by the ethics and research committee of this institution under the protocol number 3417060816.

Five brands of lubricant eyedrops purchased from the Brazilian market, namely Artelac^®^ (Bausch & Lomb Inc.), Lacrima Plus^®^ (Novartis Biociências S/A), Ecofilm^®^ (Latinofarma Indústrias Farmacêuticas Ltda), Lacribell^®^ (Latinofarma Indústrias Farmacêuticas Ltda), and Lacrifilm^®^ (União Química Farmacêutica Nacional S/A) were evaluated in this study (Table 1).

**Table 1:** The lubricating eyedrops analyzed and their active ingredients

Product	Active compound
Lacrifilm®	Sodium Carboxymethyl Cellulose 5 mg/mL
Ecofilm®	Sodium Carboxymethyl Cellulose 5 mg/mL
Lacribell®	Dextran 1 mg/mL, Hypromellosis 3 mg/mL
Lacrima® Plus	Dextran 1 mg/mL, Hypromellosis 3 mg/mL
Artelac®	Cetrimide 1 mg/mL + Hypromellosis 3.2 mg/mL

To assess whether there is a reference standard for the mass of the lubricant eyedrop solutions as a function of their volume, five samples of 20 ± 0.02 µL of the five brands studied were captured using an adjustable micropipette (Eppendorf, 20 µL) and weighed using a calibrated precision scale (Bioprecisa Electronic Balance FA2104N) with a resolution of 0.001g. The eyedrop bottles were labeled A-E and randomly selected for measurement. The global mean mass of all weighed drops was used as a reference to evaluate the potential discrepancy of individual eyedrop volume.

Analyses of variance (one-way ANOVA) were performed using Sigma Stat software (SYSTAT, San Jose, California). Statistical significance was set at a threshold of *p*&lt;0.05.

## RESULTS

**Figure 1:**
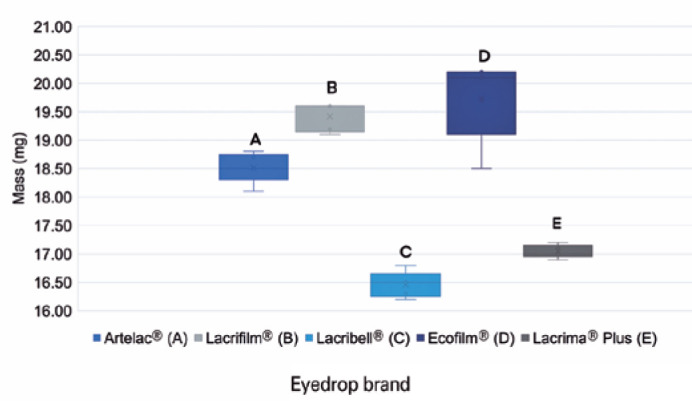
Mass distribution of 20 _µ_L samples of each of the five brands of lubricant eyedrops (N=25).

The individual mass of each 20 µL sample is shown in table 2, and the mass distribution of each drop is visualized in figure 1. The global mean mass of all weighed drops was 18.24 mg.

**Table 2:** Measurements of individual drop masses of five brands of lubricant eyedrops available on the Brazilian market

Sample #	Artelac^®^ (A)	Lacrifilm^®^ (B)	Lacribell^®^ (C)	Ecofilm^®^ (D)	Lacrima^®^ Plus (E)
1	18.7 mg	19.1 mg	16.2 mg	18.5 mg	17.0 mg
2	18.8 mg	19.2 mg	16.5 mg	20.1 mg	17.0 mg
3	18.5 mg	19.6 mg	16.3 mg	20.2 mg	17.2 mg
4	18.1 mg	19.6 mg	16.5 mg	19.7 mg	17.1 mg
5	18.5 mg	19.6 mg	16.8 mg	20.2 mg	16.9 mg
Mean (mg)	18.5 mg	19.4 mg	16.5 mg	19.7 mg	17.0 mg
Standard Deviation (mg)	0.2 mg	0.2 mg	0.2 mg	0.6 mg	0.1 mg

Among the different manufacturers, significantly different values of mass for a drop with volume of 20 µL were recorded (*p*&lt;0.001). However, the mass variation among samples from the same product was not statistically different.

**Figure 2:**
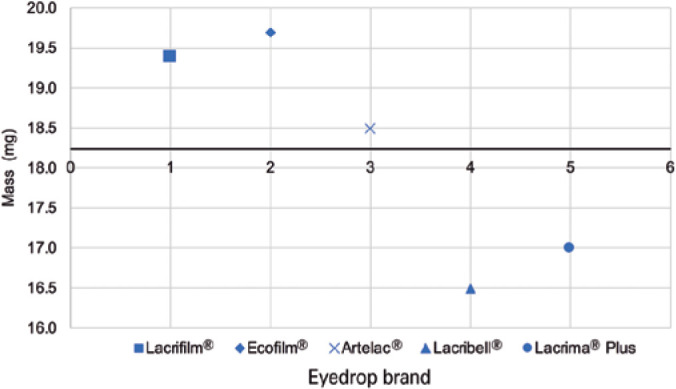
Mean drop mass differences of the five samples from each manufacturer in comparison to the global mean of 18.24 mg (central line).

The difference in the mean drop mass of the five samples from each manufacturer, compared to the global average of 18.24 mg, is shown in figure 2. Heterogeneity was noted in drop mass across products in comparison to the global mean.

## DISCUSSION

Minimizing discrepancies in drop volume is important in maintaining pharmaceutical equivalence of topically-applied agents. Our study identified significant variations in the drop volumes of five pharmaceutically equivalent common brands of lubricant eyedrops currently available on the Brazilian market, as well as considerable heterogeneity in drop volume in comparison to a global mean mass of 18.24 mg. These variations were likely due to factors such as the physicochemical characteristics of each eyedrop solution, as well as the viscosity, density, and surface tension of the agent. Indeed, the American Pharmacopoeia states that manufacturers should account for these characteristics and calibrate the dispenser appropriately for each preparation, allowing for a variation of up to 15% in the volume of each drop^(8)^. Additionally, the Brazilian National Health Surveillance Agency (ANVISA) notes the fundamental importance of administering the correct dose of a medication, since under-dosing can be ineffective and over-dosing can lead to unwanted or adverse side effects^(24)^.

The possibility that products with the same concentration of active ingredients may present with the same mass in a predetermined volume of 20 µL was considered. However, brands with the same composition and concentration of active ingredients (Lacrifilm^®^ and Ecofilm^®^; and Lacribell^®^ and Lacrima^®^ Plus) showed close, but still significantly different, mass mean values. Despite variation across different formulations, the samples from different manufacturers individually presented reliable mean and standard deviation drop masses. This finding suggests that, when developing a new topical agent, manufacturers may use this simple methodology to compare the mass of an ideal eyedrop of 20 µL and the mass of one actual drop measured directly from the eyedrop bottles being tested. The difference between the ideal drop volume of that product and the real drop volume can be used to adjust factors such as the bottle dropper design, nozzle tip design, or the physicochemical characteristics of the product. A pre-established reference measure may lead to the production of more appropriately sized eyedrops for use in human eyes.

The variable eyedrop volumes among different manufacturers suggest that a more extensive study of parameters involving not only the physical properties of the contained solution, but also the position and force used to dispense each eyedrop, may be warranted. This variation is possibly accentuated when using non-standard dropper bottles, implying that a generic or equivalent drug may not have the same drop volume despite having the same concentration of active ingredients in the solution. Moreover, despite the benefits afforded by a controlled laboratory environment, the current study was limited by the use of standard laboratory equipment, which does not account for external patient factors, such as the level of force or instillation angle used to dispense the drop. As patient manipulations are less predictable, our methodology sought to maintain consistency by using simple equipment to ensure repeatability and reliability.

The results of this study reveal a significant variability in drop volume, and therefore dosage, of standard topical ocular medications, bearing significant implications for standardization across manufacturers. Even for products with the same concentration of active ingredients, it is important to conduct specific tests for each product and adjust dosage discrepancies when appropriate. Weighing the mass of 20 µL eyedrop samples is a simple methodology to establish a reference pattern for the drop volume of a specific dispenser-solution drop. Maintaining consistency in dosage delivery is essential in delivering high-quality formulations and ensuring appropriate treatment for patients who rely on topical medications for sight-threatening eye diseases.
